# Enhanced Upconversion Emission from K^**+**^‑Modified NaGdF_4_:Er^3**+**^/Yb^3**+**^ Particles in Flexible Free-Standing Films for Thermal Sensing Application

**DOI:** 10.1021/acsomega.5c07064

**Published:** 2025-10-31

**Authors:** Ana Beatriz Acosta, Vitor dos Santos de Souza, Francisco Recco Torres, Yaman Masetto Nicolai, Luiz Fernando dos Santos, José Maurício Almeida Caiut, Rogéria Rocha Gonçalves

**Affiliations:** † Laboratório de Materiais Luminescentes Micro e Nanoestruturados — Mater Lumen, Centro de Nanotecnologia E Engenharia Tecidual-CNET, Departamento de Química, FFCLRP, 124588 Universidade de São Paulo, Ribeirão Preto, São Paulo 14040-901, Brazil; ‡ Grupo de Nanomateriais E Sistemas Luminescentes- nanolum, Departamento de Química, FFCLRP, Universidade de São Paulo, Ribeirão Preto, São Paulo 14040-901, Brazil

## Abstract

Rare earth-based upconversion (UC) luminescent materials have potential use in optoelectronics, bioimaging, and thermal sensing. In this study, K^+^-modified NaGdF_4_:Er^3+^/Yb^3+^ nanoparticles, embedded in transparent and flexible carboxymethyl cellulose-based free-standing films, exhibited enhanced UC luminescence and highly sensitive thermal sensing. Incorporating K^+^ ions into the NaGdF_4_:Er^3+^/Yb^3+^ particles modified their rod-like morphology without altering their hexagonal crystalline phase, intensified their UC emission, and prolonged their excited-state lifetimes. Under 980 nm excitation, rising K^+^ content in the NaGdF_4_:Er^3+^/Yb^3+^ particles prolonged the lifetimes of the Er^3+^ ^4^S_3/2_ (538 nm) and ^4^F_9/2_ (653 nm) → ^4^I_15/2_ transitions from 54.6 to 308.6 μs and from 208.7 to 691.0 μs, respectively. In addition to enhanced UC emission intensity, the incorporation of K^+^ also influences the branching ratios of emission pathways, thereby altering the relative intensities of green and red emissions and increasing the ^4^S_3/2_ → ^4^I_15/2_ transition intensity. Systematic evaluation of the temperature-dependent luminescence response of the K^+^-modified NaGdF_4_:Er^3+^/Yb^3+^ nanoparticles embedded in the polymeric films demonstrated that the resulting films can potentially function as efficient optical thermometers as the films presented high thermal sensitivity, excellent repeatability over multiple temperature cycles, and stable performance before and after they were embedded with the nanoparticles. These findings underscore that incorporating an alkali metal into a rare earth-doped material optimizes the luminescent and thermometric performance of the material, allows the material to be well dispersed in a polymeric film, and enables the preparation of free-standing films with potential use in advanced optical thermometry and multifunctional sensing.

## Graphical abstract

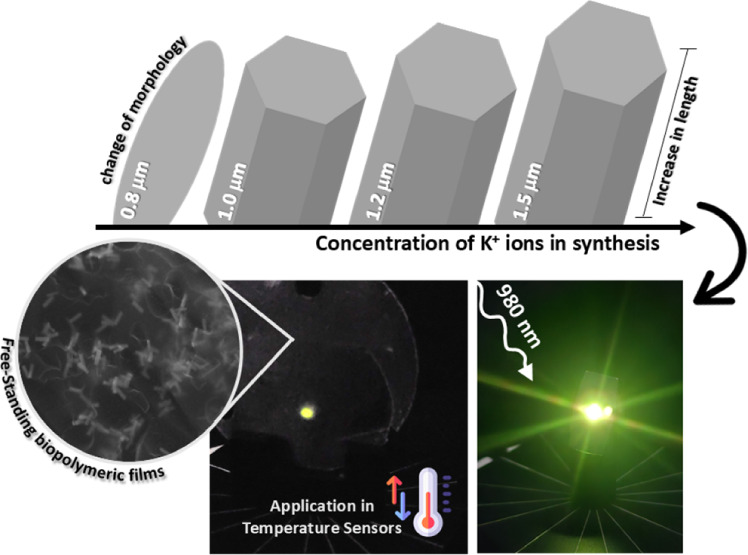

## 1 Introduction

The interest of the scientific community in developing multifunctional optical materials has increased; such materials have potential applications in strategic fields such as healthcare, environmental monitoring, energy, telecommunications, and security. In this context, rare earth-doped materials stand out for their unique luminescent properties, including sharp emission bands, long excited-state lifetimes, and high photostability. However, achieving optimal performance requires selecting a host matrix that ensures high optical transparency in the emission region of lanthanide ions, high solubility of the doped material in the solid network, low phonon energy (to minimize nonradiative losses), and robust chemical and thermal stability (to ensure long-term performance). These properties are key for enhancing the luminescence efficiency and allow rare earth-based materials to be seamlessly integrated into advanced optoelectronic and photonic devices.^1^

Among the host matrixes described in the literature, rare earth fluorides, such as REF_2_, REF_3_, REF_4_, and AREF_4_ (where RE = rare earth and A = alkali), have garnered attention due to their low phonon energy (<500 cm^–1^) when compared to oxides.^2,3^ Low phonon energy combined with the high solubility of rare earth ions allows efficient emission processes to occur in the visible and near-infrared (NIR) regions through upconversion (UC) and downshifting mechanisms following NIR excitation.^4,5^

Despite the inherent advantages of these fluoride matrices, dispersing rare earth-doped nanoparticles into polymeric systems often reduces luminescence.^6^ The luminescence quenching may significantly restrict the practical application of these materials, both under ambient conditions and upon thermal elevation, thereby posing additional challenges to the thermometric performance of the films.^7^ To mitigate this effect, efforts have been made to enhance the luminescence of rare earth ions and to improve overall performance.^7^ To counteract this effect, several strategies have been explored, and one of the most effective has been incorporating alkali metal ions, e.g., Li^+^ and K^+^, into the host matrix.^8^ Previous studies have demonstrated that alkali metal incorporation not only enhances UC emissions but also modifies particle morphology at both the nanometric^9,10^ and micrometric scales.^11^

When it comes to developing multifunctional devices, dispersing luminescent particles in polymers such as carboxymethyl cellulose (CMC) is particularly promising. CMC is widely used across various industries, including the biomedical, pharmaceutical, textile, food, cosmetics, and packaging sectors. CMC is odorless, tasteless, noncaloric, and physiologically inert, which makes it an excellent thickener, emulsion stabilizer, adhesive, and moisture binder.^12–14^ In the pharmaceutical field, CMC, hydrogels, and organic–inorganic hybrid films have been extensively employed to control drug release, to emulsify drugs, and to stabilize bioactive compounds^15–17^ given that they are biocompatible, highly stable, and sensitive to pH, not to mention that they can interact with active compounds such as drugs and enzymes. In particular, CMC plays a crucial role in numerous biomedical applications, including tissue engineering,^18,19^ wound dressings,^20,21^ fabrication of three-dimensional scaffolds for biocompatible implants, and development of artificial organs or substitutes for polymeric extracellular matrices.^22–24^ These characteristics make CMC an attractive medium for integrating rare earth-doped materials into flexible, transparent, and biocompatible platforms.

UC luminescent materials are also being investigated for sensing purposes, especially luminescence-based thermometry. Flexible and stretchable sensors with multiple sensing functions are in high demand for applications in healthcare, environmental monitoring, and industrial processes. Traditional temperature measurement methods often require direct physical contact, which can be limiting in sensitive or inaccessible environments. Thermometry based on luminescence has thus emerged as an efficient alternative that enables temperature to be remotely sensed with high precision, sensitivity, and spatial resolution.^25,26^ Among luminescent thermometers, those based on temperature-dependent variations in the luminescence properties of rare earth ions are noteworthy. These thermometers are attractive due to their versatility, chemical stability, narrow emission bands, high quantum yields, and long excited-state lifetimes.^27^

In this study, we have fabricated free-standing polymer composite films embedded with K^+^-modified NaGdF_4_-based luminescent nanoparticles and optimized the structural and luminescent properties of the resulting films. We show that K^+^ incorporation enhanced the UC emission of the particles and improved their thermometric performance. Evaluation of the temperature-dependent luminescence response of the particles embedded in polymeric films or not demonstrated that they can potentially function as highly sensitive optical thermometers. Our findings highlight the potential of these multifunctional materials for advanced applications in optical sensing and thermal monitoring.

## 2 Experimental Section

### 2.1 Synthesis of Er^3+^/Yb^3+^ Co-Doped Na_1–*x*_K_*x*_GdF_4_ Particles

The 5 mol % Er^3+^, 20 mol % Yb^3+^:Na_1–*x*_K_*x*_GdF_4_ particles were synthesized via a hydrothermal method assisted by ethylenediaminetetraacetic acid (EDTA; LABSYNTH, analytical grade). Aqueous solutions of the dopant nitrates were prepared by dissolving the respective oxides (Yb_2_O_3_ and Er_2_O_3_; Sigma-Aldrich, 99.9%) in concentrated nitric acid (HNO_3_, Sigma-Aldrich, 65% by weight) under stirring at 70 °C for 3 h. After drying, the resulting solutions were diluted with deionized water.

The reaction mixture was assembled in a 25 mL Teflon-lined reactor by combining aqueous solutions of Er^3+^ and Yb^3+^ nitrates, gadolinium­(III) nitrate hexahydrate (Gd­(NO_3_)_3_·6H_2_O; Sigma-Aldrich, 99.9%), and alkali metal (sodium and/or potassium, with NaNO_3_/KNO_3_ molar ratio changing as reported in Table 1) nitrates (NaNO_3_ and/or KNO_3_; Sigma-Aldrich, ≥99.0%). Separately, 1 mmol of EDTA was dissolved in 5 mL of deionized water under stirring, while 10 mmol of ammonium fluoride (NH_4_F; Sigma-Aldrich, 98.0%) was dissolved in 5 mL of deionized water. Both solutions were added dropwise to the reactor containing rare earth and alkali metal precursors. The total volume was adjusted to 20 mL, and the mixture was stirred for 20 min.

**Table 1:** Nominal Na^+^ and K^+^ Molar Quantity Used to Synthesize 5 mol % Er^3+^, 20 mol % Yb^3+^ Co-Doped Sodium Gadolinium Fluoride by the Hydrothermal Methodology

Sample Label	NaNO_3_/KNO_3_ molar ratio	K^+^ content in the synthesis (%)^a^
N1K0	1:0	0
N1K1	1:1	16.7
N2K3	2:3	25.0
N0K1	0:1	33.3

a Considering the molar amounts of Na^+^, originating from disodium EDTA and NaNO_3_, and K^+^, originating from KNO_3_, added during the hydrothermal synthesis.

The sealed Teflon reactor was placed in an autoclave and was heated in a furnace at 185 °C for 15 h. Upon completion of the reaction, the suspension was cooled to room temperature, and the nanoparticles were recovered by centrifugation. The resulting wet powder was thoroughly washed with distilled water and ethanol and dried at 80 °C for 24 h under ambient conditions

#### 2.1.1 Preparation of CMC/GPTMS Free-Standing Films Containing N0K1 Particles

The CMC/GPTMS (GPTMS = 3-glycidoxypropyl)­trimethoxysilane) films were obtained by the casting method. The N0K1 particles were dispersed in water by using an ultrasonic bath to prepare two films, F1 and F2. For F1, 10 mL of water was used to dissolve CMC and GPTMS (Acros Organics, 97%) at a 1:1:0.1 CMC/GPTMS/N0K1 mass ratio. The mixture was subjected to stirring and heating until the components were completely dispersed. For F2, the same volume of water, 10 mL, was used; however, the amount of CMC and GPTMS was reduced by half, while the 1:1:0.1 CMC/GPTMS/N0K1 mass ratio was maintained. The mixture was stirred until it was fully homogenized. Then, the F1 or F2 formulation was poured into Petri dishes and left to dry at room temperature to give the free-standing films F1 and F2 (Figure 1).

**Figure 1:**
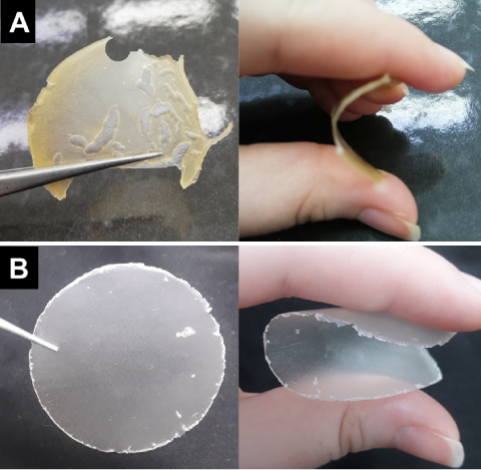
Images of free-standing films obtained from formulations (A) F1 and (B) F2.

### 2.2 Synthesis of Eu^3+^ Doped Na_1–*x*_K_*x*_GdF_4_ Particles

In addition to the material codoped with Er^3+^ and Yb^3+^, hexagonal Na_1–*x*_K_*x*_GdF_4_ containing Eu^3+^ was synthesized for Raman measurements to avoid luminescence from the Er^3+^ ions under laser excitation. The 25 mol % Eu^3+^ doped Na_1–*x*_K_*x*_GdF_4_ particles were synthesized via a hydrothermal method assisted by ethylenediaminetetraacetic acid (EDTA; LABSYNTH, analytical grade) at 185 °C for 15 h, following a similar procedure already described in Section 2.1. The same variation in the NaNO_3_/KNO_3_ molar ratio was used (as reported in Table 2). Aqueous solutions of the dopant nitrate were prepared by dissolving the respective oxide (Eu_2_O_3_, Sigma-Aldrich, 99.9%) in concentrated nitric acid (HNO_3_, Sigma-Aldrich, 65% by weight) under stirring at 70 °C for 3 h, as presented in Section 2.1.

**Table 2:** Nominal Na^+^ and K^+^ Molar Quantity to Synthesize 25 mol % Eu^3+^ Doped Sodium Gadolinium Fluoride by the Hydrothermal Methodology

Sample Label	NaNO_3_/KNO_3_ molar ratio	K^+^ content in the synthesis (%)^a^
N1K0-Eu	1:0	0
N1K1-Eu	1:1	16.7
N2K3-Eu	2:3	25.0
N0K1-Eu	0:1	33.3

a Considering the molar amounts of Na^+^, originating from disodium EDTA and NaNO_3_, and K^+^, originating from KNO_3_, added during the hydrothermal synthesis.

### 2.3 Characterization

X-ray diffraction data were collected using a Siemens-Bruker D5005 diffractometer. The equipment operated with CuKα radiation (1.5418 Å), a graphite monochromator, and a step size of 0.02°. The diffractograms were obtained in the 2θ range from 10° to 90°. Raman spectra were collected by employing a high-performance XploRA PLUS micro-Raman spectrometer (HORIBA France SAS) operating with a 638 nm red laser. Raman scattering spectra were obtained from powder samples homogeneously distributed on a glass slide. Analyses were performed with a × 50 VIS-LWD-DF objective lens, and the spectra were recorded from 150 to 750 cm^–1^. Sample surface was analyzed by scanning electron microscopy (SEM); a Prisma E SEM microscope (Thermo Scientific) was employed. The powder samples were placed on a carbon tape, and images were captured at magnifications of 30,000× and 45,000×, at 20 keV. Fourier-transformed infrared spectroscopy (FTIR) measurements were performed by mixing the powders with KBr pellets and recording the spectrum on a PerkinElmer Spectrum Two FT-IR spectrometer from 4000 to 400 cm^–1^.

The rheological measurements of the CMC-based gels were conducted on an MCR52 rheometer (Anton Paar). Parallel plate geometry (diameter = 35 mm), as well as a temperature of 22.0 °C, adjusted by a recirculating water bath, was employed. In the oscillatory analyses, the samples underwent an amplitude sweep, with the amplitude increasing from 0.5% to 1000% at a constant angular frequency of 1.0 rad/s, or a frequency sweep, with the frequency varying from 1.0 to 100.0 rad/s at a fixed amplitude of 1.0%. During rotational analyses, viscosity was varied by means of the flow sweep test, with a shear rate ranging from 1.0 to 100.0 s^–1^.

Photoluminescence analyses were performed at room temperature using a Horiba Scientific Fluorolog 3 spectrofluorometer. An FL-1073 detector was used for the visible region, and an uncooled H10330-75 detector with a 22.5° detection was employed for the infrared region. The UC spectra of the samples were obtained by applying a 980 nm CW diode laser (CrystaLaser, DL980) with power ranging from 50 to 450 mW. Temperature-dependent UC spectra were recorded at temperatures ranging from 293 to 373 K. To this end, the sample was placed in a platinum crucible inside a LINKAM Scientific temperature control chamber (T95-HT). The temperature was allowed to stabilize for a few minutes before each measurement was accomplished. A 976 nm continuous-wave fiber laser (DMC—Laser Tool), coupled to an optical fiber, was used as the excitation source; the power density was 6.56 W·cm^–2^. The spectra were acquired by coupling an optical fiber to the detector of the spectrofluorometer (the same equipment used for photoluminescence measurements).

## 3 Results and Discussion

Figure 2 presents the X-ray diffractograms of the Na_*x*_K_1–*x*_GdF_4_: 5 mol % Er^3+^, 20 mol % Yb^3+^ particles (*x* = 0, 0.5, 0.75, and 1) synthesized herein. The peaks at 17.2°, 29.8°, 30.4°, 34.5°, 39.1°, 46.1°, 51.1°, and 53° confirm that the pure hexagonal structure of NaGdF_4_ emerged in all of the samples (JCPDS 01-080-8787). It is important to highlight that NaGdF_4_ can crystallize in either cubic (*Fm*3̅*m* space group) or hexagonal structures, with phase control being particularly challenging at the nanoscale and critically affecting the transition probabilities and luminescence efficiency. In this work, all synthesized samples exhibited the X-ray diffraction pattern corresponding to the hexagonal phase, attesting to the phase purity of the particles obtained by hydrothermal synthesis at 185 °C for 15 h, which is the most favorable for optical applications. According to the literature, there are three space groups for the hexagonal phase: P6̅, P6̅2m, and *P*6_3_/*m*. Space groups P6̅ and P6̅2m converge to a common structure characterized by sodium ions 9-fold coordinated (CN = 9). In contrast, space group *P*6_3_/*m* bears sodium atoms distributed into six coordination sites.^28^

**Figure 2:**
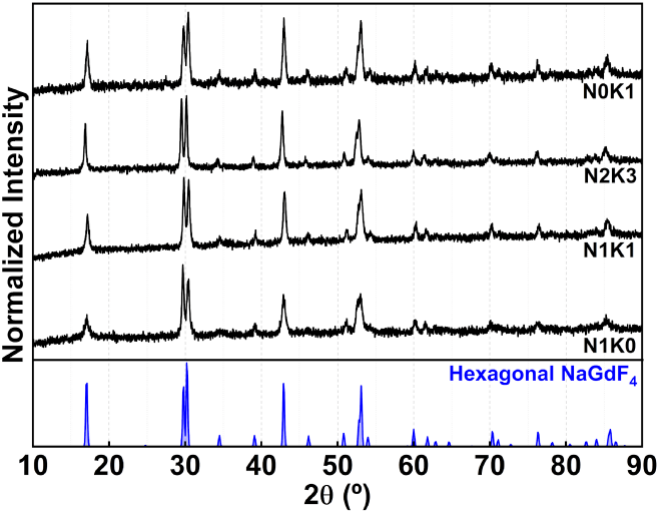
X-ray diffractograms of Na_1–*x*_K_*x*_GdF_4_ particles doped with 5 mol % Er^3+^ and 20 mol % Yb^3+^ for *x* = 0, 0.5, 0.75, and 1, labeled as N1K0, N1K1, N2K3, and N0K1, respectively. The X-ray diffraction patterns of hexagonal NaGdF_4_ (light blue) standard are presented for comparison.

Burns (1965) was the first to detail the crystallographic structure of the P6̅ space group. In this configuration, fluoride anions (F^–^) are arranged in triangular patterns, while the cations occupy three types of sites: one site is fully occupied by a lanthanide ion (Ln^3+^), one site is statistically partially occupied by 1/2 Na^+^ and 1/2 Ln^3+^, and one site is statistically occupied by Na^+^ and vacancies and exhibits point group symmetry C_3_.^29^

The *P*6_3_/*m* space group, in turn, contains two cationic sites. The first site is fully occupied by Na^+^ and has point symmetry C_3i_, while the second site, with point symmetry C_3h_, is randomly filled with Na^+^ and Ln^3+^. However, the sites with point symmetry C_3h_ may undergo structural distortions, assuming C_s_, C_3_, or C_1_ symmetries.^29,30^

Finally, space group P6̅2m has two distinct cationic sites: one site has D_3h_ point symmetry and is fully occupied by Ln^3+^, whereas the other site exhibits C_3h_ point symmetry and is statistically filled with 3/4 Na^+^ and 1/4 Ln^3+^.^30^ Based on the literature, NaLnF_4_ (Ln = La, Ce, Pr, Sm, Eu, Gd) crystals are isostructural and adopt the hexagonal P6̅ (P6h) space group.^2^

Although X-ray diffraction did not reveal any significant changes in the crystalline structure of the samples, confirming that all remain isomorphic, scanning electron microscopy evidenced a pronounced modification in rod formation. Figure 3 shows the SEM images of N1K0, N1K1, N2K3, and N0K1 particles, where the rods display increasingly well-defined hexagonal shapes and longer lengths (0.8, 1.0, 1.2, and 1.5 μm, respectively) with increasing KNO_3_ concentration in the synthesis.

**Figure 3:**
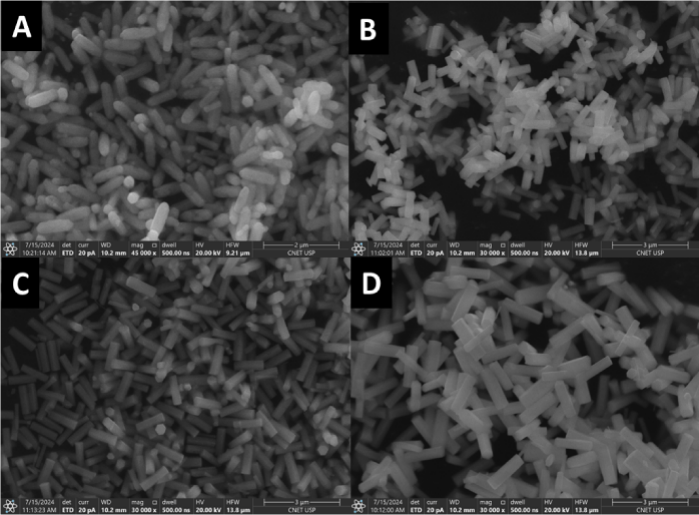
Scanning Electron Microscopy (SEM) images of (A) N1K0, (B) N1K1, (C) N2K3, and (D) N0K1.

According to the literature, high K^+^ concentrations promote the growth of hollow rods, which feature smooth, flat surfaces as well as open, cracked, and sharp ends. On the other hand, low K^+^ concentrations cause filled hexagonal rods to grow.^11^ Accordingly, the morphology observed in these samples supports the low incorporation of K^+^, despite the absence of NaNO_3_ in the synthesis of the N0K1 sample, since all compositions already contain a significant amount of Na^+^ originating from disodium EDTA used as the complexing agent.

Figure 4 shows the Raman scattering spectra of the N1K0–Eu, N1K1–Eu, N2K3–Eu, and N0K1–Eu samples, measured on similar samples doped with 25 mol % Eu^3+^ to avoid luminescence from the Er^3+^ ions under laser excitation. The group factor analysis for hexagonal P6̅ NaGdF_4_ predicts a total of 25 Raman active modes, as stated in Table 3 in accordance with the literature.^31^

**Figure 4:**
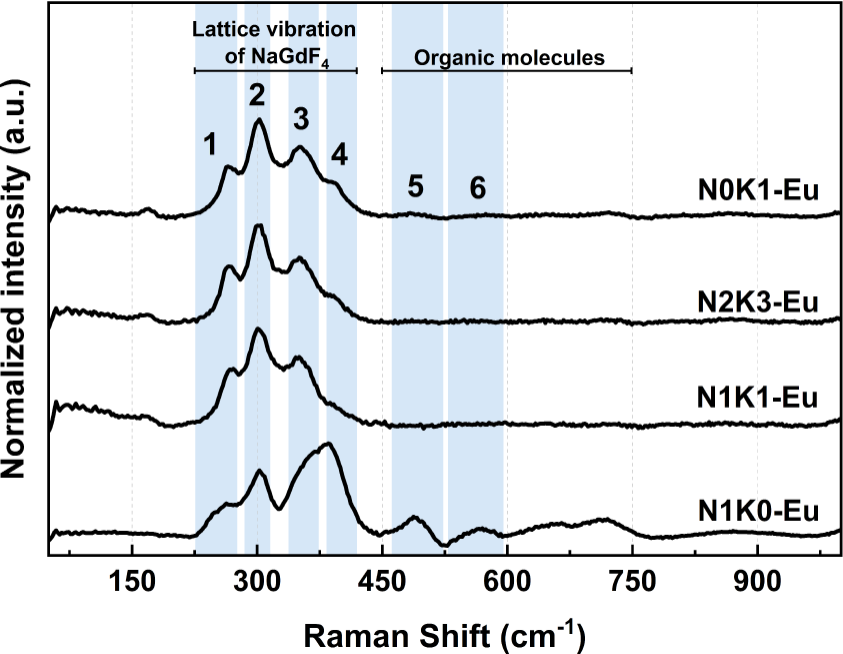
Raman scattering spectra of N1K0-Eu, N1K1-Eu, N2K3-Eu, and N0K1-Eu samples.

**Table 3:** Group Factor Analysis for NaGdF_4_ Compounds Belonging to the Hexagonal P6̅ Space Group^a^ ^b^

Atom	Wyckoff Site	Symmetry	Irreducible representation
NaGdF_4_ – Hexagonal P6̅, (174), C_3h_^6^			
Gd	1a	C_3h_	1 ^1^E’ + 1 ^2^E’
Gd	1f	C_3h_	1 ^1^E’ + 1 ^2^E’
Na	1f	C_3h_	1 ^1^E’ + 1 ^2^E’
Na	2h	C_3_	1 A’ + 1 ^1^E’ + 1 ^1^E″ + 1 ^2^E’ + 1 ^2^E″
F	3j	C_1v_	2 A’ + 2 ^1^E’ + 1 ^1^E″ + 2 ^2^E’ + 1 ^2^E″
F	3k	C_1v_	2 A’ + 2 ^1^E’ + 1 ^1^E″ + 2 ^2^E’ + 1 ^2^E″

a Γ_Total_ = 5 A’ + 8 ^1^E’ + 3 ^1^E″ + 8 ^2^E’ + 3 ^2^E″, Γ_Acoustic_ = A″ + ^1^E’ + ^2^E’.

b Γ_Raman_ = 5 A’ + 7 1E’ + 3 1E″ + 7 2E’ + 3 2E″ – 25 active modes.

The most intense bands, located between 200 and 400 cm^–1^, and summarized in Table 4, are assigned to the vibrational modes of the NaGdF_4_ host lattice, consistent with previous reports in the literature.^2^ Vibrational modes observed above ∼400 cm^–1^ are typically attributed to organic groups present on the nanoparticle surface.^32,33^ The spectral profile of the N1K0-Eu sample, characterized by three dominant peaks within 200 and 400 cm^–1^ (maximum at 265.1, 302.6, and 352.4 cm^–1^) closely resembles those reported for the hexagonal NaLnF_4_ structure with space groups P6̅, and *P*6_3_/*m*.^2^ Notably, the peak at ∼352 cm^–1^ appears overlapped with vibrational modes extending up to 450 cm^–1^ (maximum at ∼385 cm^–1^), in addition to other bands at 487 and 565 cm^–1^. This observation suggests that in the absence of potassium particle growth not only results in distinct morphologies but also leads to the presence of organic groups on the nanoparticle surface. Confirmation of this interpretation can be found in Figure S3, in which the FTIR spectra reveal intense absorption bands assigned to the stretching vibrations of carboxylic −COOH groups in the 1700–1550 cm^–1^ region. In addition, characteristic stretching modes of the O–H and N–H groups are observed at approximately 3300 and 3500 cm^–1^, respectively, while bands around 1700 and 1600 cm^–1^ are indicative of the C=O vibration. Among these features, the broad band centered at 3442 cm^–1^ is attributed to O–H stretching, whereas the doublet at 1610 and 1401 cm^–1^ corresponds to the asymmetric and symmetric stretching vibrations of the −COO^–^ group.^34–36^

**Table 4:** Assignment of the Main Bands in the Raman Spectra of Na_1–*x*_K_*x*_GdF_4_ (*x* = 0, 0.5, 0.75, and 1) Particles

Sample	ν_1_ (cm^–1^)	ν_2_ (cm^–1^)	ν_3_ (cm^–1^)	ν_4_ (cm^–1^)	ν_5_ (cm^–1^)	ν_6_ (cm^–1^)
N1K0-Eu	263.0	302.6	368.9	385.4	487.4	565.8
N1K1-Eu	267.1	300.6	348.3	-	-	-
N2K3-Eu	267.1	300.6	352.4	-	-	-
N0K1-Eu	265.1	302.6	352.4	-	-	-

As presented in Table 3, in the hexagonal structure (P6̅), Gd^3+^ ions occupy the 1a and 1f Wyckoff positions, both with a coordination number (CN) of 9, while the 1f site is shared statistically with 1/2 Na^+^ ions. The 2h sites (CN = 6) are half-occupied by Na^+^ and sodium vacancies (V_Na^+^_), introducing intrinsic structural defects. These include local symmetry breaking around Na^+^ ions and potential partial occupancy of 2h sites by Ln^3+^ ions, contributing to the broadening of Raman bands and being linked to variations in luminescence efficiency. Additionally, the incorporation of K^+^ and Eu^3+^ dopants may further perturb the lattice by partially occupying the 1f or 2h positions. Given the ionic radius for K^+^ 1.38 Å (CN 6) and 1.55 Å (CN = 9),^37^ these substitutions are expected to induce local distortions and shifts in Raman spectra, consistent with previous reports involving the substitution of Ln^3+^ ions in NaLnF_4_ compounds.^2^ This shift indicates a potential decrease in the effective phonon energy of the material, according to eq 1:

1 $$E_{h}=\frac{\underset{i}{\sum }E_{i}w_{i}A_{i}}{\underset{i}{\sum }w_{i}A_{i}}$$

where *E*_*i*_, *w_i_*, and *A_i_* represent the position, full width at half-maximum (fwhm), and relative intensity of each Lorentzian subband, respectively. However, due to the low levels of K^+^ substitution, the main vibrational modes’ characteristics of hexagonal crystalline NaGdF_4_ with P6̅ symmetry are only slightly affected. According to the literature, variations in vibrational features may arise from the substitution of different rare-earth ions and from morphological changes, even when the same hexagonal crystalline structure is preserved.^38,39^ In our case, the most pronounced effects are associated with the vibrational modes of the organic groups on the particle surface. Notably, for samples synthesized with higher concentrations of K^+^ ions, these surface organic groups, which are responsible for luminescence quenching, are significantly reduced.

Phonons play a crucial role in the upconversion luminescence (UCL) process, particularly in energy transfer upconversion (ETU) mechanisms. When phonon energies decrease due to structural modifications or confinement effects, the rate of phonon-assisted nonradiative relaxation is reduced. This can lead to an increase in the population of excited states, enhancing the upconversion efficiency, as will be discussed further herein.

Rheological analyses of the CMC/GPTMS-based films helped to show how the F1 and F2 formulations and the presence of the K^+^-modified NaGdF_4_:Er^3+^/Yb^3+^ particles interfered with the structure. In the amplitude sweep, the point at which the curves intersected decreased (Figure 5A). At this point, the elastic modulus (*G*’), which concerns the solid component of the film, becomes less dominant and intersects with the viscous modulus (*G*”), which becomes dominant.^40^ Given that a gel is characterized by *G*’ > *G*”, this is the point where the structure breaks. This occurred at 11.9% for the films without particles and 3.0% for the films embedded with the particles. In other words, the films embedded with the nanoparticles lost their predominantly elastic structure when smaller strains were applied compared to the films without the particles, which provides evidence that the three-dimensional network became somewhat disaggregated.

**Figure 5:**
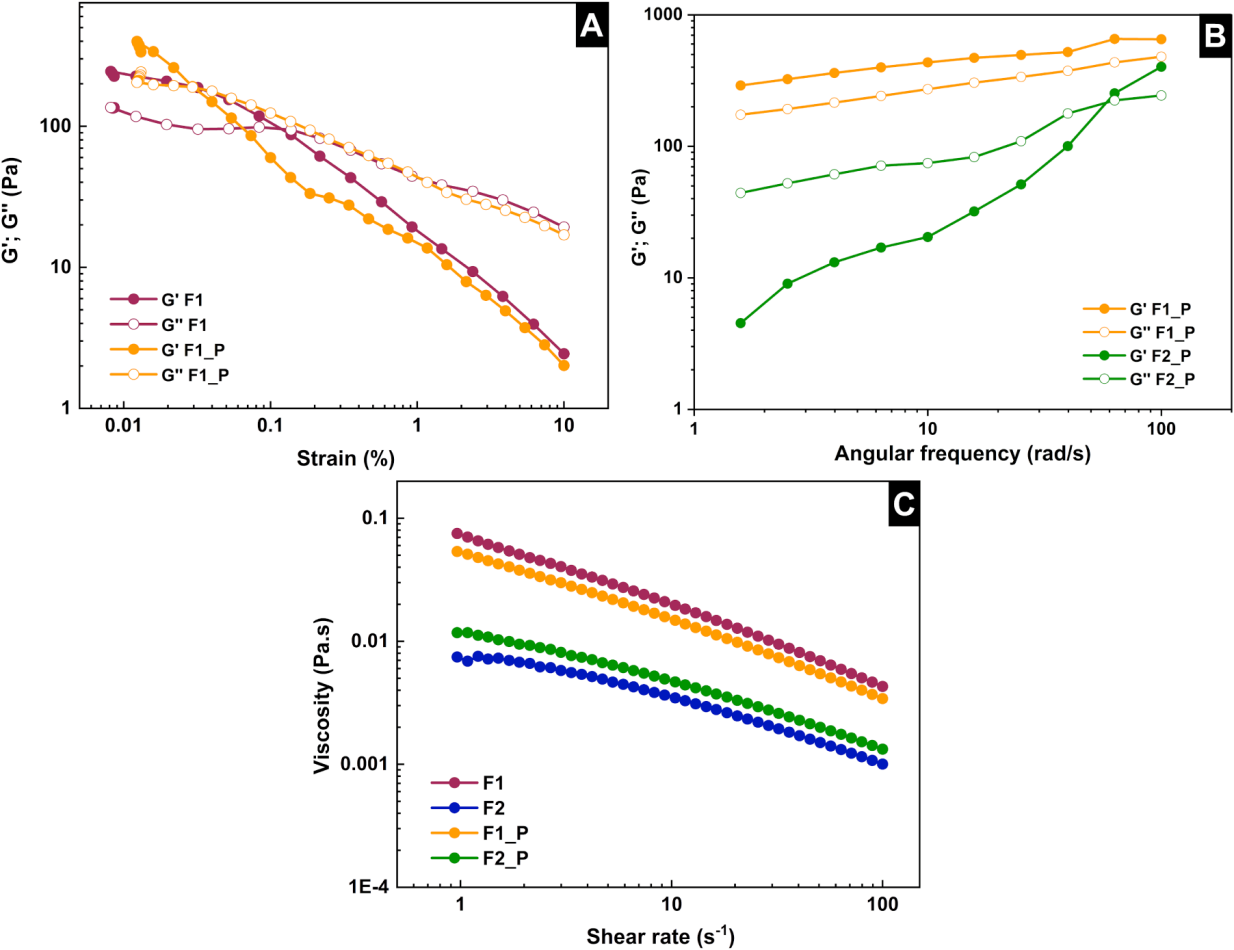
Rheograms of F1 and F2 embedded with K^+^-modified NaGdF_4_:Er^3+^/Yb^3+^ particles (P) or not: behavior of *G*’ and *G*″ along the (A) amplitude sweep and (B) frequency sweep, and (C) viscosity as a function of the shear rate.

In the frequency sweep, the viscoelastic behavior of the two formulations differed considerably (Figure 5B). F1 presented *G*’ > *G*” with increasing behavior and parallel curves, which indicates a structured gel, as previously reported.^41^ In contrast, *G*″ predominated in F2 in most of the range, and unstable moduli prevented it from being defined as a gel.

Therefore, both F1 and F2 were at the concentration threshold for gel formation; F1 can be used when the gel is required, and F2 may be better suited for obtaining films, as we did here by casting. The CMC concentration can be adjusted. CMC concentrations greater than 3% (w/v) can give a film with higher viscoelastic moduli for specific applications, such as the 3D printing of the gel by extrusion. In both F1 and F2, viscosity decreased linearly with increasing shear rate, which characterizes a pseudoplastic behavior^42^ (Figure 5C). F1 had a higher viscosity than F2, but the formulations embedded with the nanoparticles did not differ from the corresponding formulation without the particles.

The dispersion of the particles within the films was examined by scanning electron microscopy. A representative image is shown in Figure 6, highlighting the random distribution of Na_1–*x*_K_*x*_GdF_4_ rods with a higher proportion of particles embedded in the free-standing film (F1 CMC/N0K1).

**Figure 6:**
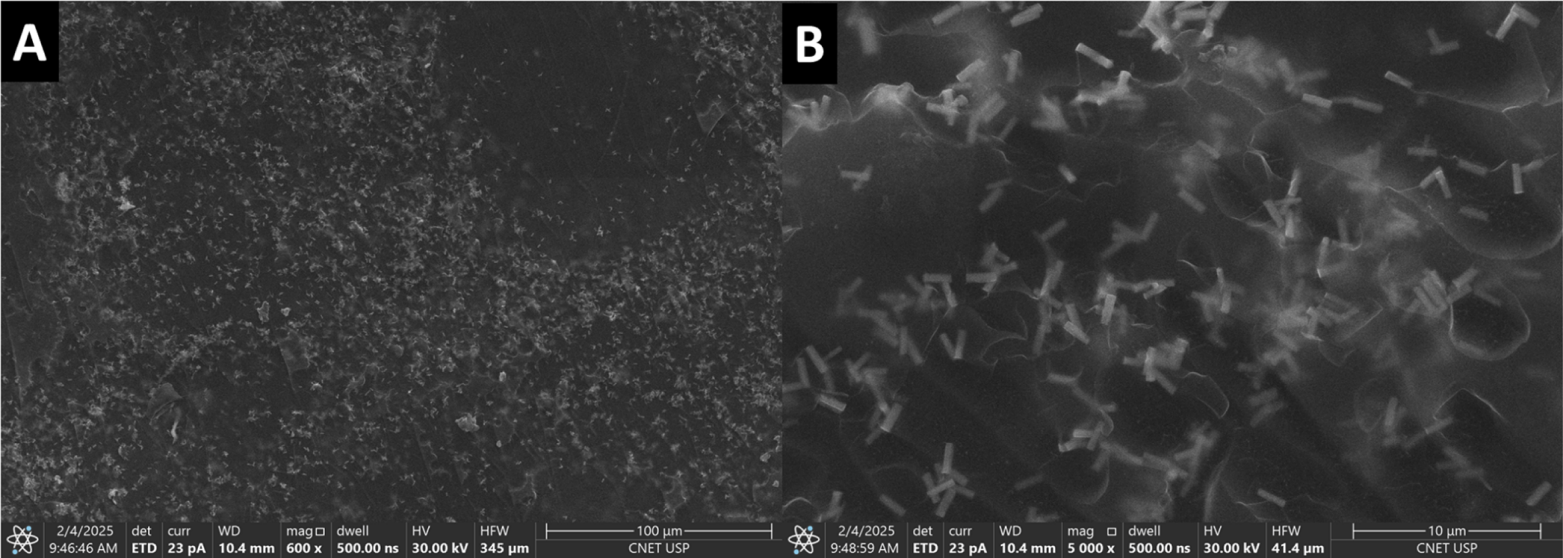
Scanning electron microscopy (SEM) images of the F1 CMC/N0K1 films at magnifications of (A) 600× – bar = 100 μm and (B) 5000× – bar = 10 μm.

We measured UC according to the setup shown in Figure 7A. Upon excitation with a 980 nm laser, the samples were emitted in the visible region (Figure 7B–E). Figure 8 shows the emission spectra in the visible region of the synthesized samples excited at 980 nm under varying excitation power. All of the spectra displayed two emission bands at approximately 525 and 540 nm, corresponding to the Er^3+^ ^2^H_11/2_, ^4^S_3/2_ → ^4^I_15/2_ transitions, which result in green light emission. Another emission band appeared at 660 nm in the red region and is characteristic of the Er^3+^ ^4^F_9/2_ → ^4^I_15/2_ transition. The emission intensified with an increasing K^+^ concentration.

**Figure 7:**
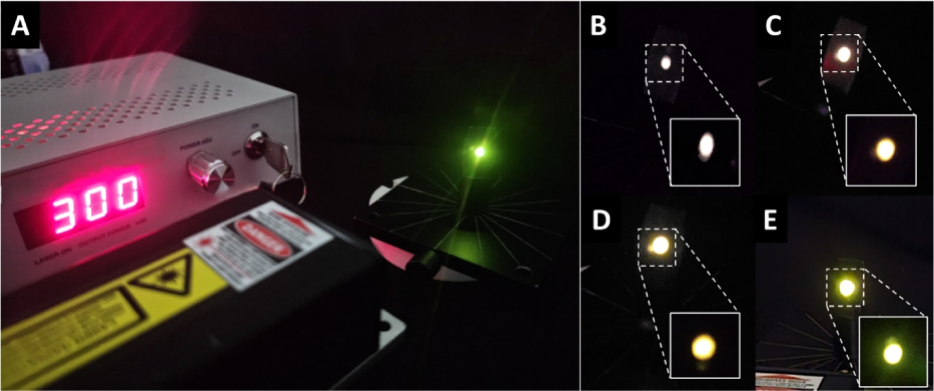
(A) Experimental setup for luminescence under 980 nm excitation (upconversion) at 300 mW power of (B) N1K0, (C) N1K1, (D) N2K3, and (E) N0K1.

**Figure 8:**
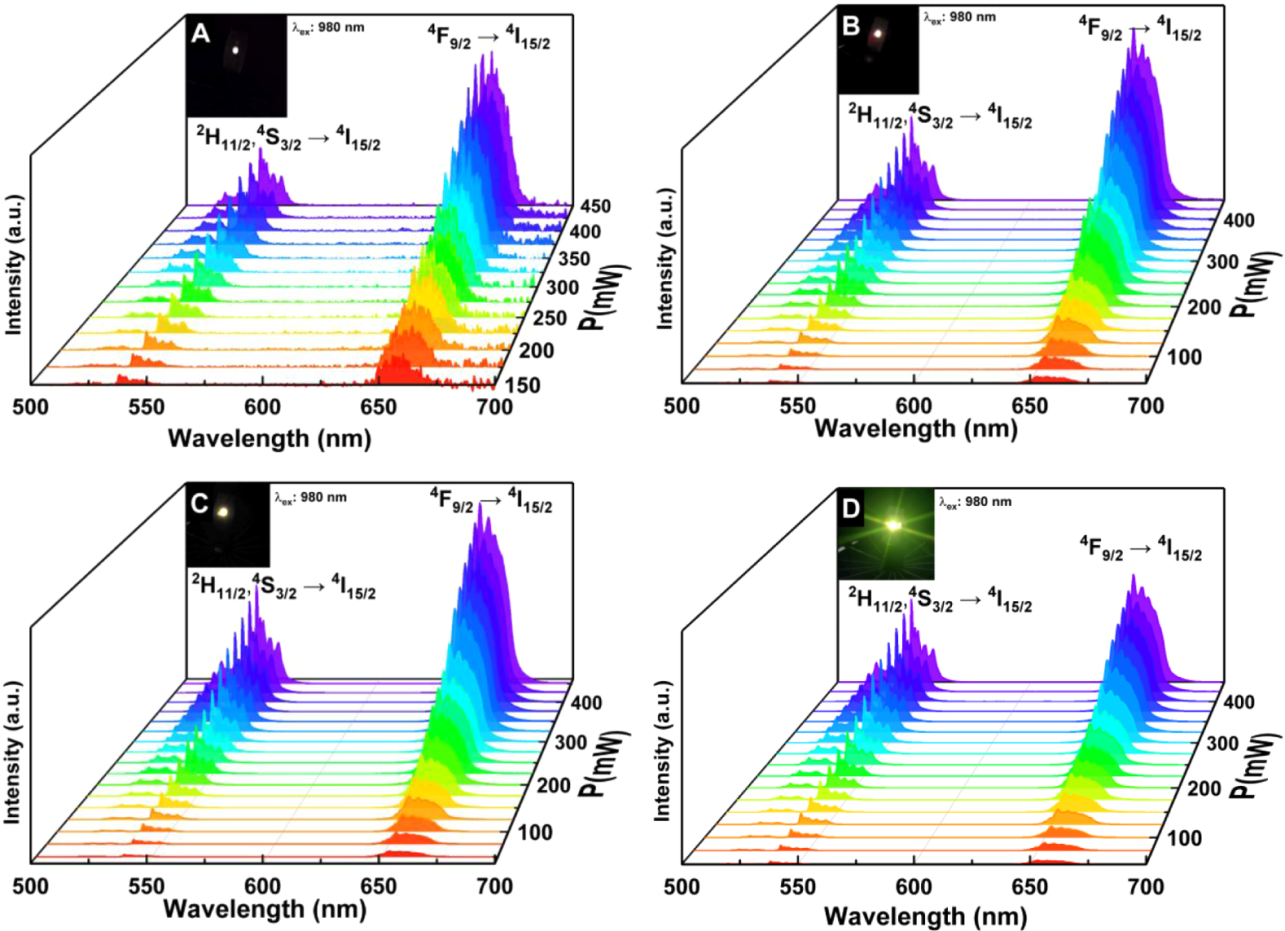
Emission spectra of samples (A) N1K0, (B) N1K1, (C) N2K3, and (D) N0K1 under excitation at 980 nm with variable laser power.

The UC luminescence is strongly influenced by variations in the transition probabilities of Er^3+^ ions, which are governed by phonon energy and local symmetry effects. The pronounced emission enhancement can be ascribed to the reduced phonon energy, as confirmed by the Raman spectra together with distortions in the local symmetry around Er^3+^ ions. Such symmetry lowering markedly increases the transition probability, leading to a significant intensification of luminescence, as clearly evidenced by the relative comparison of the emission intensities.^43,44^

On the basis of the emission intensity measurements performed with varying the laser power excitation, the number of photons involved in the UC process can be calculated. The emission intensity is directly proportional to the power raised to the number of photons involved in the process, eq 2, where *I* corresponds to the luminescence intensity, *P* is the excitation power, and *n* indicates the number of photons.^45^

2 $$I\propto P^{n}$$

The logarithmic relationship between the integrated emission intensity and the excitation power allows, through the slope of the curve, an estimation of the number of photons involved in the UC process. Figure 9 shows the log–log plots of the Er^3+^ emission intensity for the N1K0, N1K1, N2K3, and N0K1 samples corresponding to the transitions in the green and red spectral regions, as a function of the incident 980 nm laser power.

**Figure 9:**
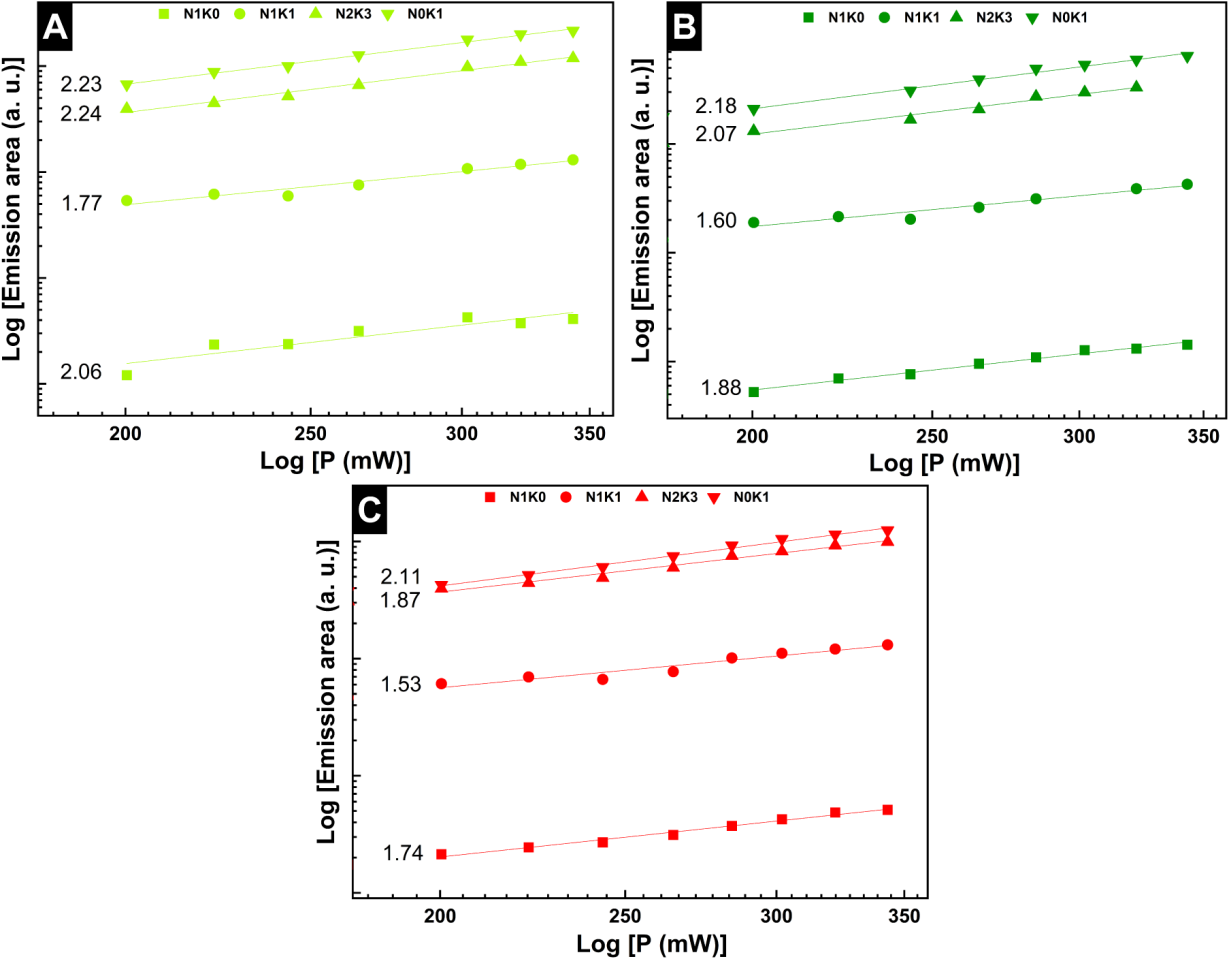
Integrated Er^3+^ emission intensity of N1K0, N1K1, N2K3, and N0K1 observed in (A) green corresponding to the ^2^H_11/2_ → ^4^I_15/2_, (B) green corresponding to the ^4^S_3/2_ → ^4^I_15/2_, and (C) red corresponding to the ^4^F_9/2_ → ^4^I_15/2_ as a function of the variation in the power of the 980 nm laser.

As shown in Figure 9, two photons participate in the UC mechanism of the material upon a 980 nm excitation. For the angular coefficients obtained near *n* = 2, the results align with the most efficient UC mechanism proposed by Auzel (1966), known as “Photon Addition by Energy Transfer” (APTE) or “Energy Transfer Upconversion” (ETU). In this mechanism, energy is transferred between sensitizer ions (Yb^3+^) and activator ions (Er^3+^), and the former efficiently absorbs the incident radiation and transfers it to the activator, which is responsible for the emission (Figure 10). This process intensifies the emission and potentially increases the overall efficiency.^46^

**Figure 10:**
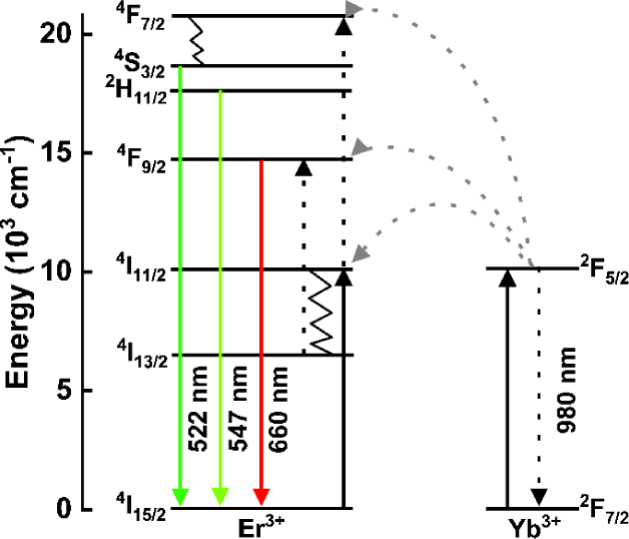
Energy transfer mechanism between the sensitizer ion (Yb^3+^) and the activator ion (Er^3+^) under 980 nm excitation.

In addition to the increase in the total emission intensity, the incorporation of K^+^ ions also influenced the ratio of emissions in the red and green regions of the samples. This modification is evidenced by the chromaticity diagrams (Figure 11), which show a shift in the coordinates from orange (580 nm) toward green (560 nm), passing through yellow (570 nm) as the K^+^ ion concentration increases.

**Figure 11:**
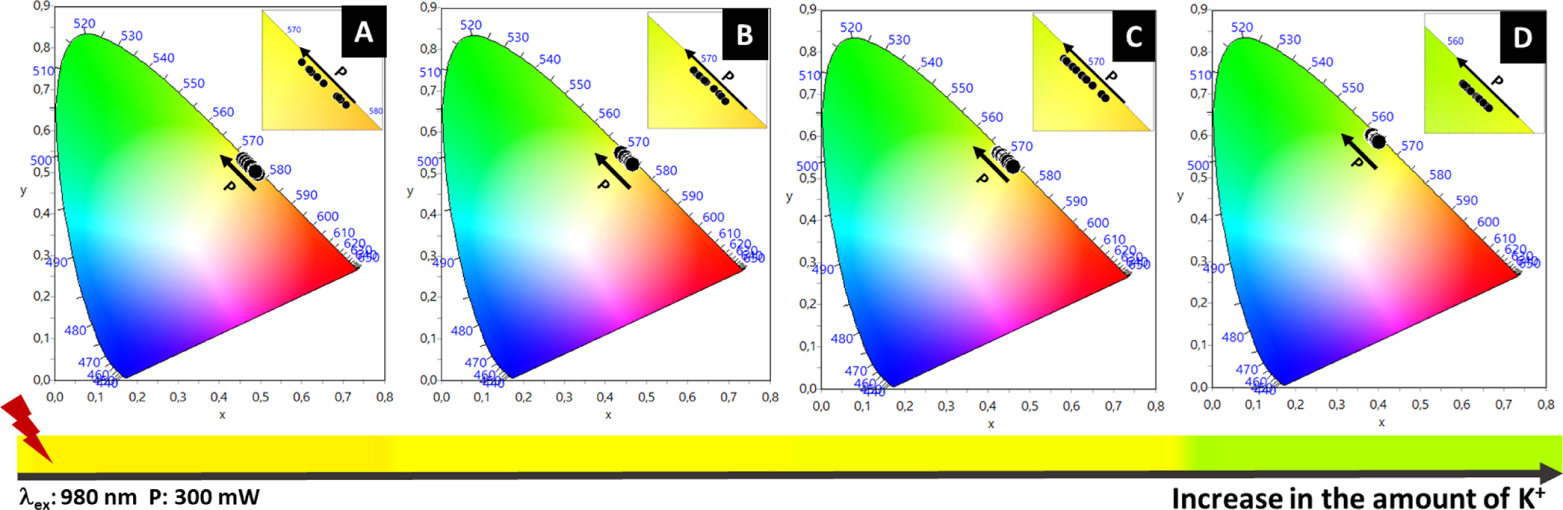
CIE 1931 chromaticity diagrams of (A) N1K0, (B) N1K1, (C) N2K3, and (D) N0K1 under 980 nm excitation with varying laser power (mW).

The emission decay curves obtained by monitoring the ^4^S_3/2_ → ^4^I_15/2_ (538 nm) and ^4^F_9/2_ → ^4^I_15/2_ (653 nm) transitions under 980 nm laser excitation (Figure 12) were fitted by using biexponential regression. The average lifetimes (τ_av_) were determined based on eq 3,^47^ by fitting the decay curves to the exponential components characterized by the times τ_1_ and τ_2_ and their respective pre-exponential coefficients *A*_1_ and *A*_2_, as presented in Table S1, Figures S1 and S2. The average lifetimes are summarized in Table 5.

**Figure 12:**
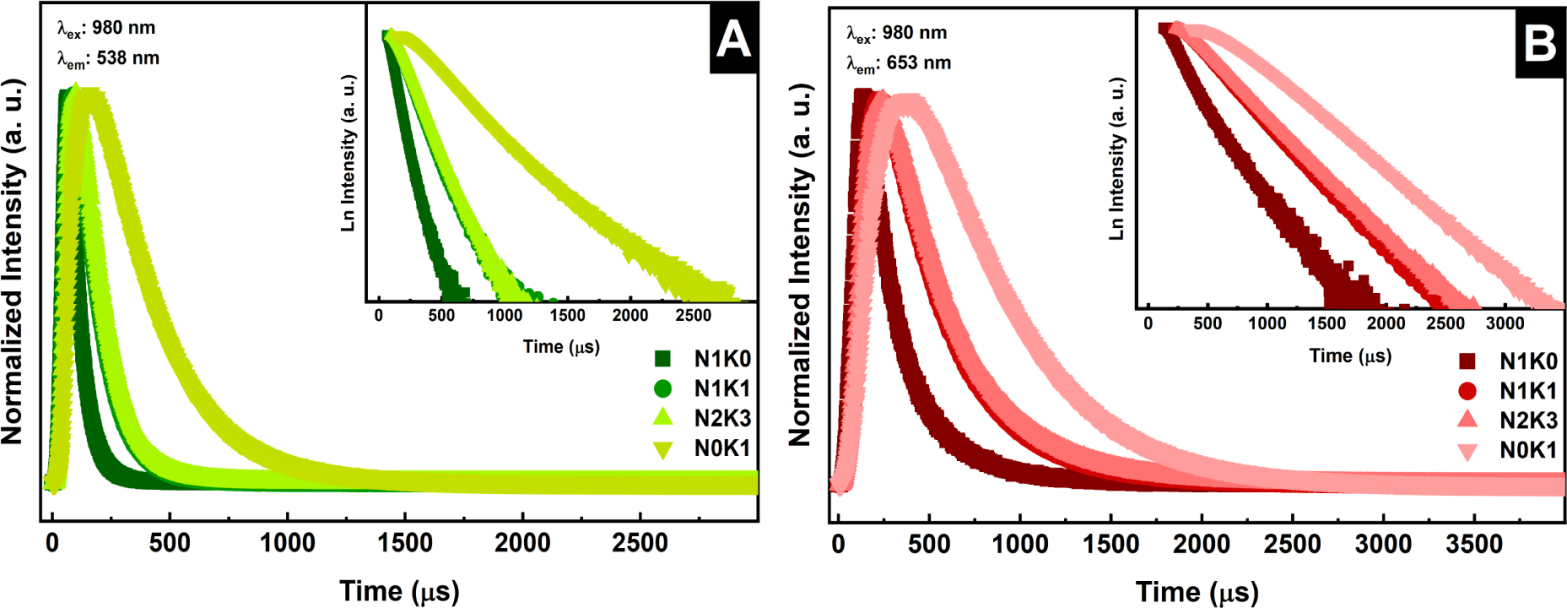
Emission decay curves of the samples obtained at (A) 538 nm and (B) 653 nm under 980 nm excitation.

**Table 5:** Lifetime Values of the Er^3+ 4^S_3/2_ and ^4^F_9/2_ Excited States Monitoring the ^4^S_3/2_ → ^4^I_15/2_ (538 nm) and ^4^F_9/2_ → ^4^I_15/2_ (653 nm) Transitions, Respectively in Samples with Different K^+^ Concentrations

	^4^S_3/2_		^4^F_9/2_	
Sample	τ (μs)	*R* ^2^	τ (μs)	*R* ^2^
N1K0	59.5	0.99986	298.0	0.99819
N1K1	113.7	0.99985	376.8	0.9999
N2K3	116.4	0.99986	400.5	0.99992
N0K1	298.4	0.99984	485.6	0.99979


3 $$\tau_{\mathrm{avg}}=\frac{\left(A_{1}\tau_{1}^{2}+A_{2}\tau_{2}^{2}\right)}{\left(A_{1}\tau_{1}+A_{2}\tau_{2}\right)}$$

Based on the data obtained, an increase in the excited-state lifetimes is observed as the K^+^ ion concentration increases. This phenomenon can be attributed to the emission decay mechanism, which extends the excited-state lifetime by suppressing competitive nonradiative processes. The reduced population of the ^4^F_9/2_ energy level, accompanied by enhanced green emission, further supports this explanation.

Previous studies involving matrices synthesized exclusively with K^+^ ions have reported significantly higher lifetimes, reaching up to 900 μs for the ^4^S_3/2_ excited state.^48^ Similarly, a study on β-NaYF_4_ with varying K^+^ concentrations (0–15 mol %) reported lifetimes of up to 472 μs for the ^4^S_3/2_ level (monitoring the ^4^S_3/2_ → ^4^I_15/2_ transition in the green region) and 785 μs for the ^4^F_9/2_ level (monitoring the ^4^F_9/2_ → ^4^I_15/2_ transition in the red region). These findings confirm that only a relatively low concentration of K^+^ ions was incorporated into the matrix, as the lifetimes observed at higher K^+^ concentrations in this work are comparable to those reported for concentrations between 7 and 10 mol % K^+^ by Liang et al. (2014).^44^

In the NaGdF_4_:Er^3+^/Yb^3+^ system, the decrease in the phonon energy, observed by Raman, contributes to the higher excited-state lifetime values, as evidenced by the luminescence decay curves (Figure 12). This suggests a reduction in multiphonon relaxation rates, which correlates with the observed enhancement in upconversion emission. Additionally, modifications in the phonon energy can influence the branching ratios of emission pathways, thereby altering the relative intensities of green and red emissions. These findings emphasize the critical role of phonon energy modulation in optimizing upconversion efficiency and highlight the importance of structural and vibrational factors in the design of luminescent materials for thermometric and optoelectronic applications.

In order to investigate the temperature sensing properties of the N0K1 particle, which exhibited the best luminescent properties, photoluminescence measurements as a function of temperature were performed before and after dispersion in the polymeric medium, covering a temperature range from 253 to 373 K. For these analyses, a power density of 6.56 W·cm^–2^ was used. Figure 13 illustrates how the intensities of the ^4^S_3/2_ → ^4^I_15/2_ (*I*_S_) and ^2^H_11/2_ → ^4^I_15/2_ (*I*_H_) transitions varied with increasing temperature for both the N0K1 sample and the corresponding polymeric film.

**Figure 13:**
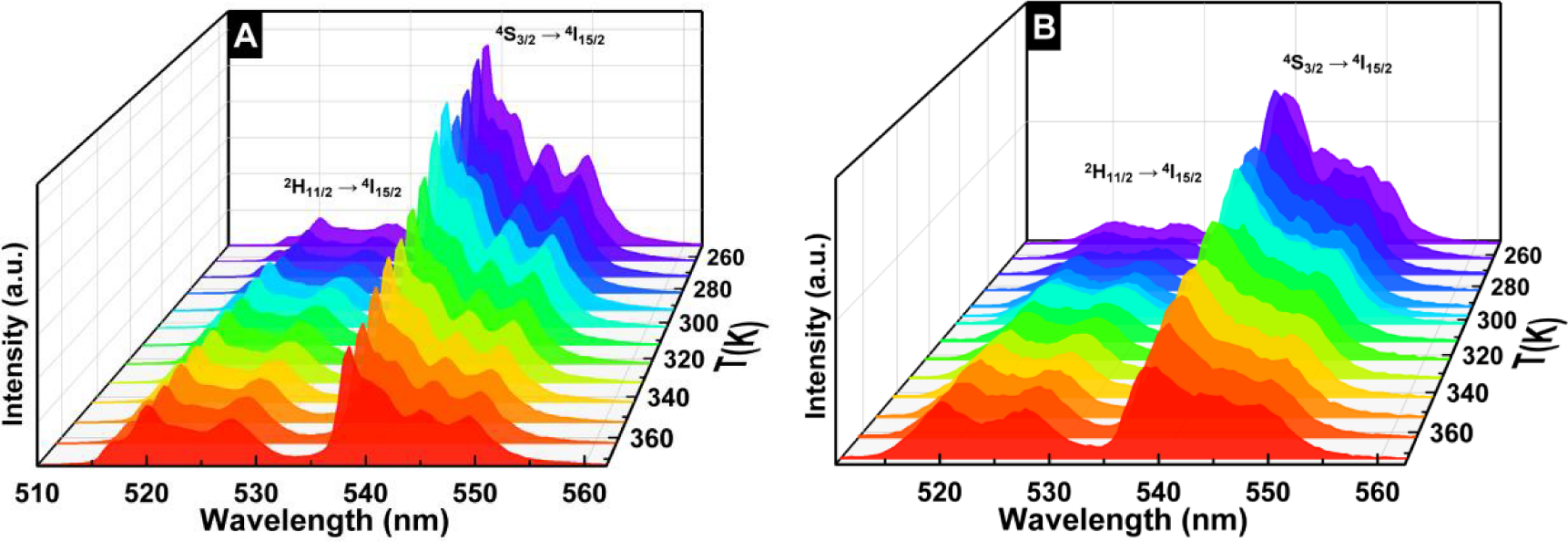
Emission spectra upon varying temperature and 980 nm excitation of (A) N0K1 and (B) N0K1 embedded in the film.

The population distribution in the two thermally coupled energy levels of Er^3+^, ^4^S_3/2_, and ^2^H_11/2_ changed as the temperature increased. Upon heating, electrons initially occupying the Er^3+^ lower-energy ^4^S_3/2_ level migrate to the higher-energy ^2^H_11/2_ level, which leads to population inversion following the Boltzmann distribution. This increase in temperature intensifies the band corresponding to the Er^3+^, *I*_H_ transition, while the band associated with the *I*_S_ transition becomes less intense.

Both N0K1 and N0K1 embedded in the film exhibited variations in the thermometric parameter (Δ, eq 4), which also increases linearly with temperature (Figure 14).^25^

4 $$\Delta =\frac{I_{H}}{I_{S}}={\mathrm{Be}}^{\frac{-\Delta E}{kBT}}$$

**Figure 14:**
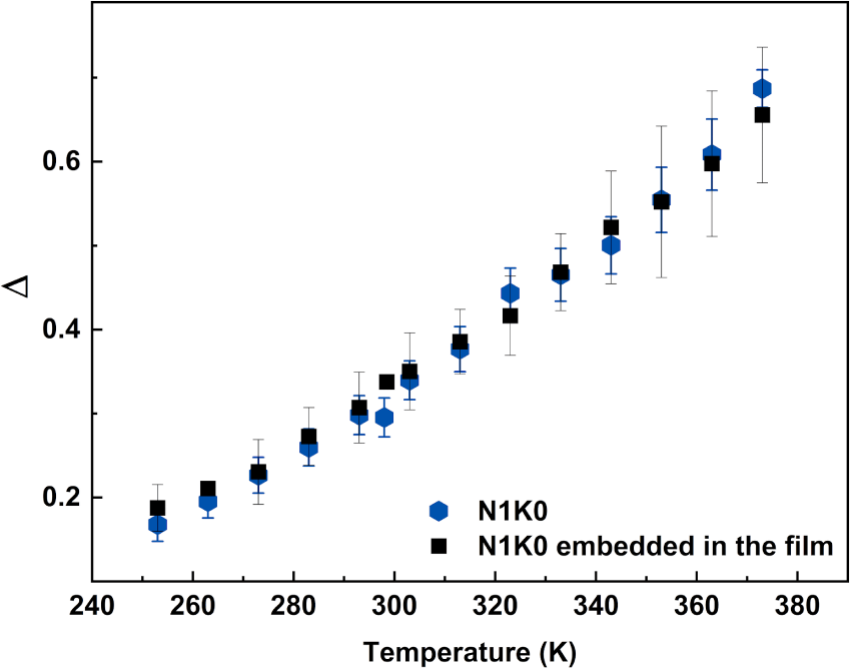
Values of the thermometric parameter Δ as a function of the sample temperature.

Additionally, we calculated the energy gap (Δ*E*, cm^–1^) by using eqs 5–7 ^25^ to obtain 672 ± 35 and 679 ± 26 cm^–1^ (Figure 15B,D), consistent with literature values of approximately 700 cm^–1^.^2^

5 E(H11/22)=∑i=1nXiAiAT

6 E(S3/24)=∑i=1nXiAiAT

7 ΔE=E(H11/22)−E(S3/24)

**Figure 15:**
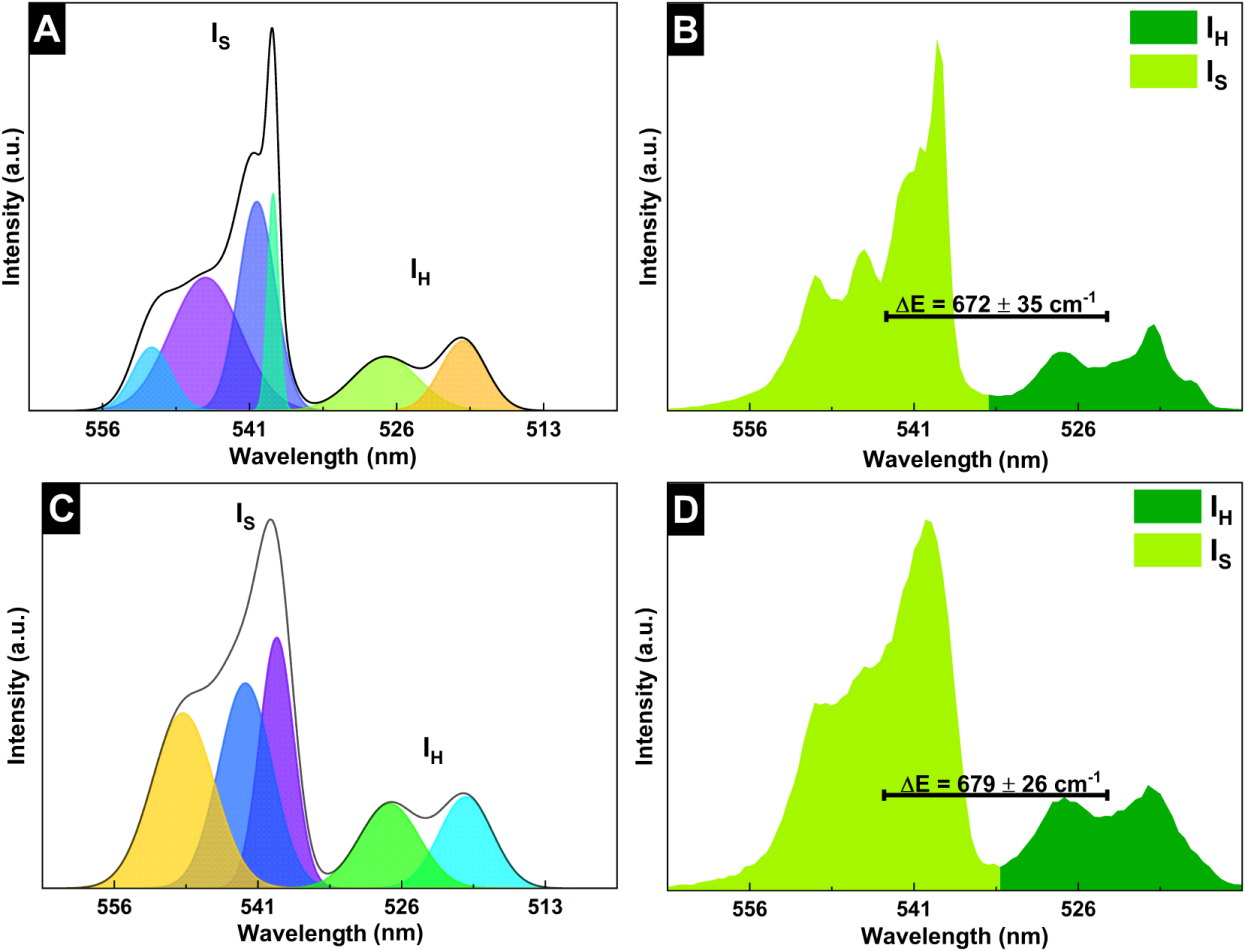
Gaussian functions (colored lines) and cumulative fit of the functions (black line) for (A) the N0K1 sample and (B) the CMC/N0K1 film. Energy difference (Δ*E*) between the thermally coupled energy levels of the Er^3+^ ions in (C) the N0K1 sample and (D) the CMC/N0K1 film.

Figure 16 reveals that for N0K1, the experimental and calculated temperatures correlated well. This confirms that N0K1 behaves as a primary thermometer,^49^ which is typical of a fluoride host lattice.^2,49^ N0K1 dispersion in the film did not affect this property, despite the higher error rates at higher temperatures. Thus, K^+^-modified NaGdF_4_:Er^3+^/Yb^3+^ particles, embedded in transparent and flexible carboxymethyl cellulose-based free-standing films, provided good results for application as a primary thermometer in temperature sensing.

**Figure 16:**
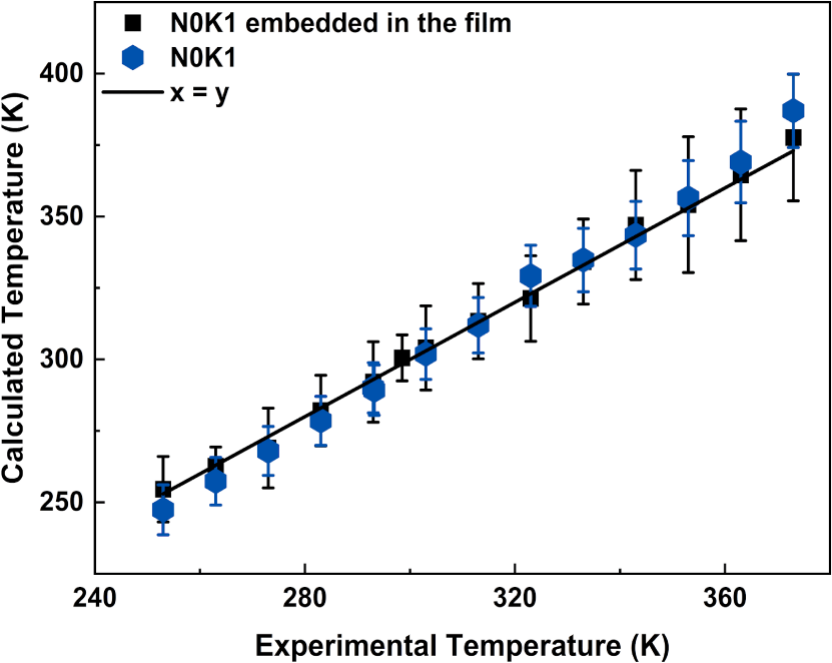
Calculated temperature as a function of the experimental temperature for N0K1 and N0K1 embedded in the film.

To evaluate the viability of this material as a primary luminescent thermometer, the relationship between the thermometric parameter (Δ) and the absolute temperature (*T*_0_) was analyzed. This relationship was directly determined by the ratio Δ/Δ_0_, incorporating the values of Δ*E*, *T*_0_, and Δ_0_, and the experimental Δ values applied in eq 8. The reference value Δ_0_ corresponded to Δ at 293.2 K for the particle and 298.5 K for the film, which represent the respective *T*_0_ temperatures. Experimental errors (Δ*T*) were calculated using eq 9

8 $$\frac{1}{T}=\frac{1}{T_{0}}-\frac{k_{B}}{\Delta E}\ln\left(\frac{\Delta }{\Delta_{0}}\right)$$

9 $$\Delta T=T^{2}\sqrt{{\left(\frac{{\delta T}_{0}}{T_{0}^{2}}\right)}^{2}+{\left(\frac{k_{B}}{\Delta E}\right)}^{2}\left[{\left(\frac{\delta \Delta E}{k_{B}}\ln\left(\frac{\Delta }{\Delta_{0}}\right)\right)}^{2}+{\left(\frac{\delta \Delta_{0}}{\Delta_{0}}\right)}^{2}+{\left(\frac{\delta \Delta }{\Delta }\right)}^{2}\right]}$$

We evaluated how the luminescent thermometer performed on the basis of the relative thermal sensitivity (Sr%) (eqs 10,11 and Figure 17C) and repeatability measurements. We obtained Δ over 10 consecutive cycles of increasing and decreasing temperature (from 273 to 323 K) (Figure 17A,B). The obtained Sr% values for N0K1 and N0K1 embedded in the film (1.06 ± 0.04% K^–1^ and 1.05 ± 0.05% K^–1^ at 303 K, respectively) are slightly lower than Sr% reported for other NaLnF_4_ systems (close to 1.20% at 300 K).^50,51^ However, N0K1 and N0K1 embedded in the film presented similar Sr% values of approximately 1.5% K^–1^ at 253 K. Moreover, Δ did not vary during the temperature cycles in either case.

10 $$S_{r}=\frac{1}{\Delta }\left|\frac{\partial \Delta }{\partial T}\right|=\frac{\Delta E}{k_{B}T^{2}}$$

11 $${\delta S}_{r}=S_{r}\sqrt{{\left(\frac{\delta \Delta E}{\Delta E}\right)}^{2}+{\left(-2\frac{\theta T}{T}\right)}^{2}}$$

**Figure 17:**
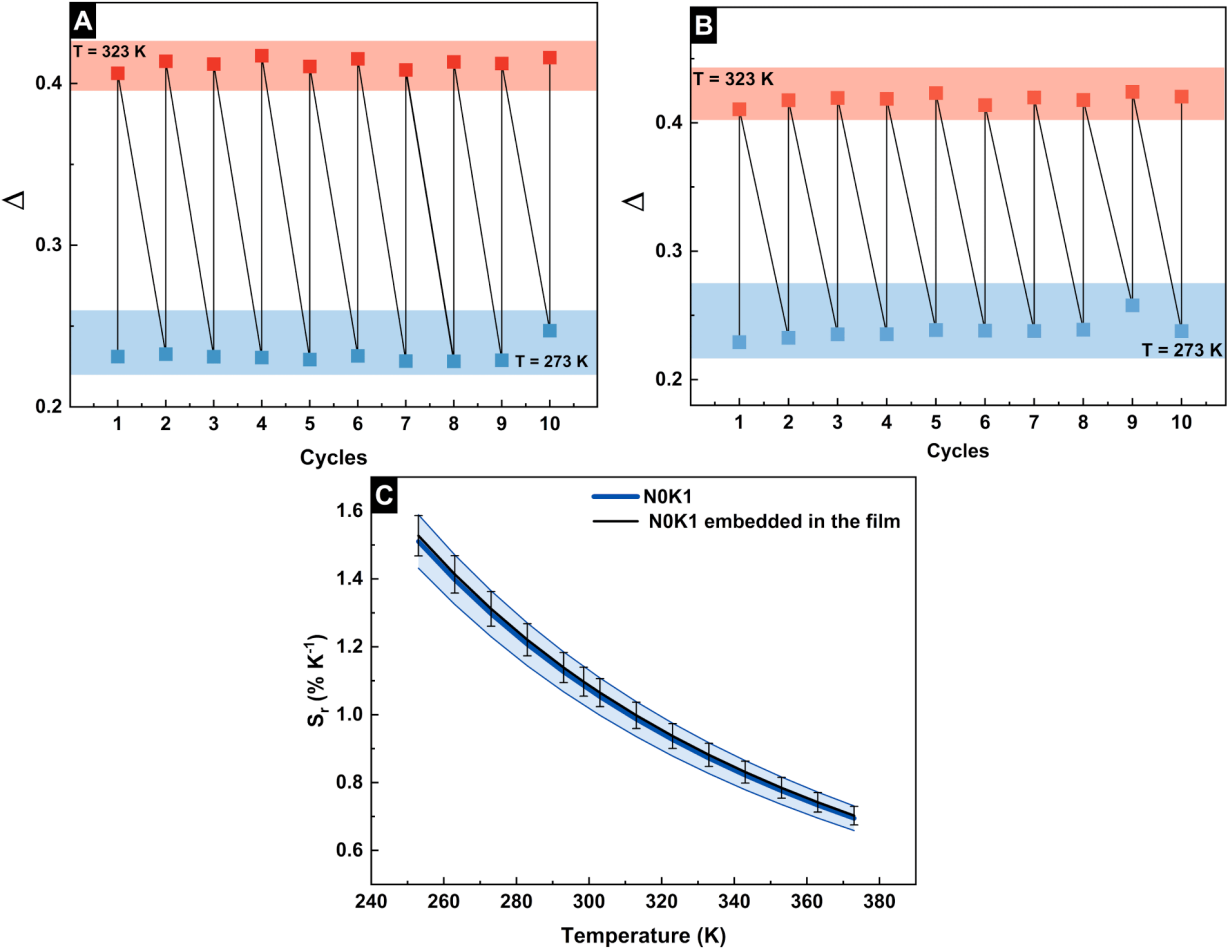
Cycling the thermometric parameter at 273 and 323 K for (A) N0K1 and (B) N0K1 embedded in the film. (C) Thermal sensitivity of N0K1 and N0K1 embedded in the film.

where θ*T* = 0.1 K, the uncertainty given by the thermocouple.

To highlight the variations in thermometric parameters and sensitivity values, Table 6 was prepared to compare the system presented in this work, both the particles and the film, with analogous systems reported in the literature.

**Table 6:** Comparative Values of Thermometric Parameters and Thermal Sensitivity in Sodium Fluoride and Lanthanide-Based Systems

Matrix	Temperature	thermometric parameter (Δ)	Sr% values (%K^–1^)	ref
NaGdF_4_: Yb^3+^/Er^3+^	303–573 K	0.2–0.7	0.21 at 491 K	^52^
NaGdF_4_: Yb^3+^/Er^3+^	303–548 K	0.2–1.1	0.43 at 523 K	^53^
NaGdF_4_: Yb^3+^/Er^3+^	300–450 K.	0.25–0.85	0.4 at 450 K	^54^
NaYF_4_:20%Yb^3+^/2%Er^3+^	160–300 K	0.0–0.25	1.20 at 300 K	^50^
NaYF_4_:Yb^3+^/Er^3+^	298–693 K	0.1–1.0	1.24 at 300 K	^51^
NaGdF_4_: 20%Yb^3+^/2% Er^3+^/15% Cr^3+^	77–500 K	0.0–0.9	0.93 at 298 K	^55^
N0K1	253–373 K	0.1–0.7	1.5 at 253 K	This work
N0K1 embedded in the film	253–373 K	0.1–0.7	1.5 at 253 K	This work

## 4 Conclusion

Incorporating K^+^ into Na_1–*x*_K_*x*_GdF_4_:5% mol Er^3+^, 20% mol Yb^3+^ particles proved to be an effective strategy for modifying the morphology of the rods without altering the obtained crystalline phase. This enhanced the luminescence properties and prolonged the lifetime of Er^3+^ transitions, making the matrix more efficient for optoelectronic applications.

Fabricating transparent and flexible films containing these luminescent nanoparticles revealed a challenge related to the nonhomogeneous dispersion of the particles within the polymeric film. However, luminescence was visible to the naked eye under excitation at 980 nm, which demonstrated the potential of these films for optical device and sensor applications.

Furthermore, K^+^-modified NaGdF_4_:Er^3+^/Yb^3+^ particles embedded in the film exhibited good correlation in temperature measurements with promising thermal sensitivity values and excellent repeatability. These findings indicate the potential of these materials for optical thermometry applications, contributing to the development of more efficient and reliable thermal sensors.

Thus, this study reinforces the importance of modifying a host matrix with K^+^ ions and incorporating luminescent particles into polymers, highlighting their potential for various technological applications, particularly in the fields of luminescent devices and temperature sensors.

## Supplementary Material



## Notes

Calculation of average lifetimes, fitting of decay curves, and FTIR measurements (PDF)
