# Supplementary material for: Enhanced Upconversion Emission from K + ‑Modified NaGdF4:Er3+ /Yb3+ Particles in Flexible Free-Standing Films for Thermal Sensing Application
Source: ACS Omega. 2025 Oct 31;10(50):61514–27. doi: 10.1021/acsomega.5c07064 (PMC12750256; doi:10.1021/acsomega.5c07064)
Supplement: Supplementary file 1 [file ao5c07064_si_001.pdf]

Enhanced Upconversion Emission from K^{+}-Modified $NaGdF_4:Er^{3+}/Yb^{3+}$ Particles in Flexible Free-Standing Films for Thermal Sensing Application

Ana Beatriz Acosta[1], Vitor dos Santos de Souza[1], Francisco Recco Torres[2], Yaman Masetto Nicolai[1], Luiz Fernando dos Santos[1], José Maurício Almeida Caiut[2] and Rogéria Rocha Gonçalves[1]

[1]Laboratório de Materiais Luminescentes Micro e Nanoestruturados — Mater Lumen, Centro de Nanotecnologia e Engenharia Tecidual- CNET, Departamento de Química, FFCLRP, Universidade de São Paulo, Ribeirão Preto, SP, Brazil

[2]Grupo de Nanomateriais e Sistemas Luminescentes- NanoLum, Departamento de Química, FFCLRP, Universidade de São Paulo, Ribeirão Preto, SP, Brazil

Corresponding author: *Rogéria Rocha Gonçalves (rrgoncalves@ffclrp.usp.br)*

Supporting Information

1. *Lifetime*

The average lifetimes (τ_{av}) were determined based on Equation S1, by fitting the decay curves to the exponential components characterized by the times τ_1 and τ_2 and their respective pre-exponential coefficients A_1 and A_2, as presented in Table S2 and Figures S1-S2.

$$\tau_{avg} = \frac{(A_1\tau_1^2 + A_2\tau_2^2)}{(A_1\tau_1 + A_2\tau_2)} \qquad \textbf{(Equation S1)}$$

Table S1. Lifetime values (τ_1 and τ_2) and pre-exponential parameters (A_1 and A_2) from the emission decay curves of the samples obtained at 538 nm ($^4S_{3/2}$) and 653 nm ($^4F_{9/2}$) under 980 nm excitation.

Samples	$^4S_{3/2}$				$^4F_{9/2}$			
	τ_1 (us)	τ_2 (us)	A_1	A_2	τ_1 (us)	τ_2 (us)	A_1	A_2
**N1K0**	48.22	125.04	0.94	0.06	140.82	337.00	0.37	0.61
**N1K1**	98.93	448.36	0.99	0.01	329.16	537.59	0.85	0.15
**N2K3**	106.46	497.25	0.99	0.01	357.14	582.96	0.87	0.13
**N0K1**	247.48	969.77	0.98	0.02	485.6	-	1.00	-

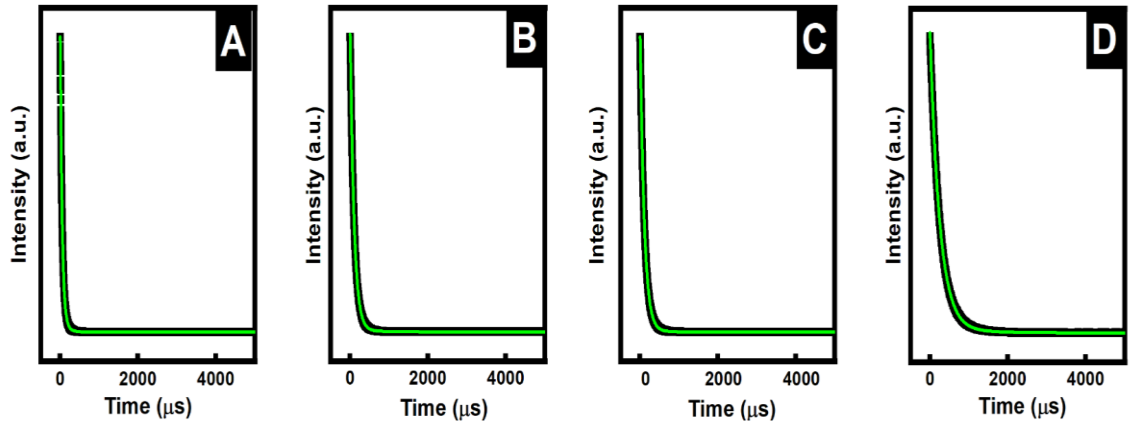

Figure S1. Fitted decay curves of samples (A) N1K0, (B) N1K1, (C) N2K3, and (D) N0K1 obtained at 538 nm under excitation at 980 nm.

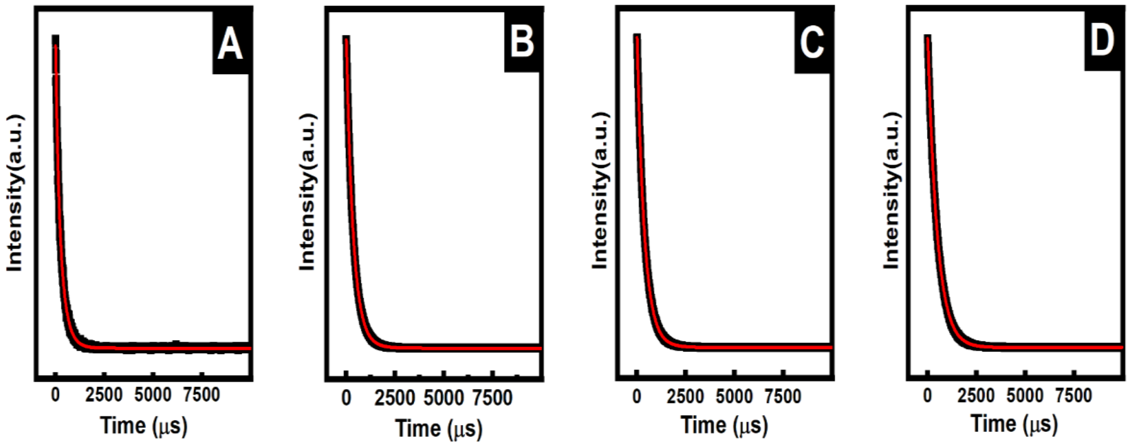

Figure S2. Fitted decay curves of samples (A) N1K0, (B) N1K1, (C) N2K3, and (D) N0K1 obtained at 653 nm under excitation at 980 nm.

2. *FTIR Measurements*

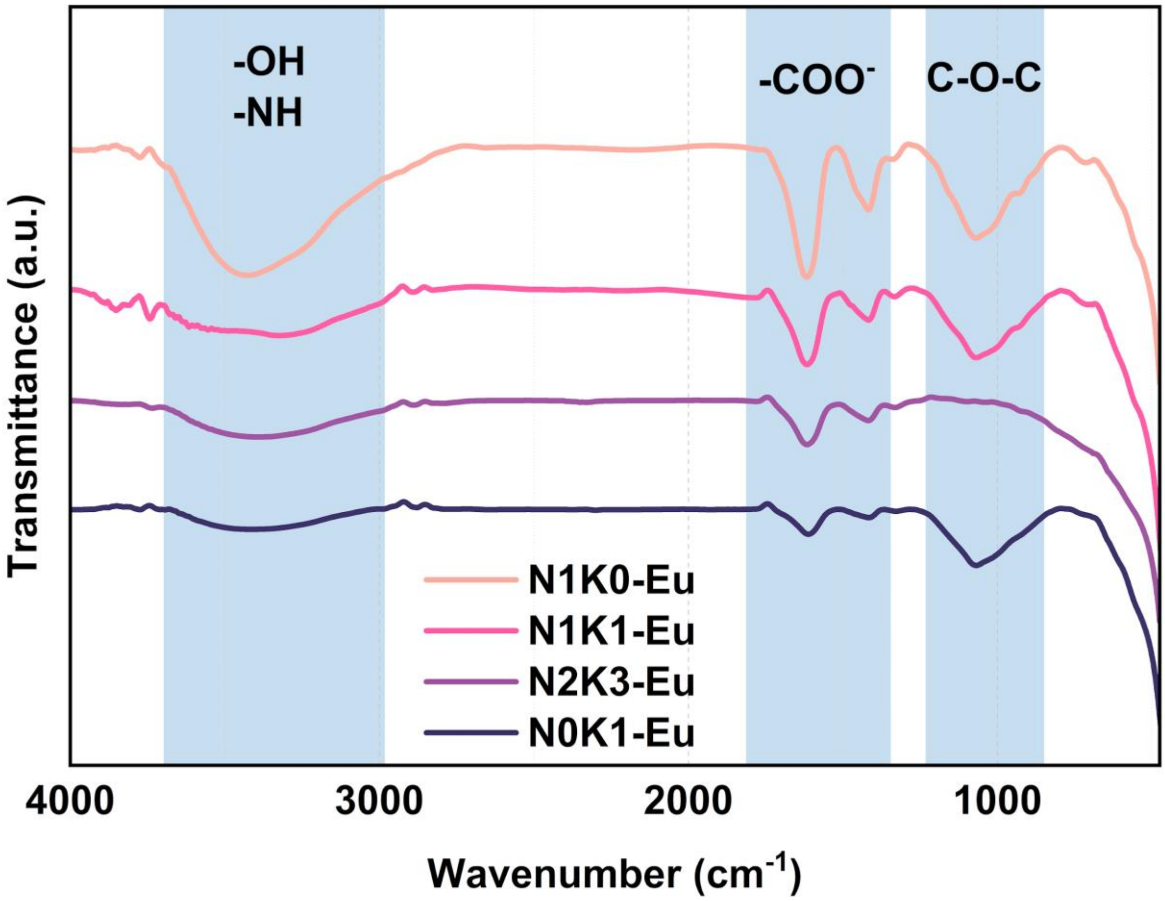

Figure S3. FTIR spectra of N1K0-Eu, N1K1-Eu, N2K3-Eu and N0K1-Eu samples.
